# Study protocol for the Vivistim GRASP registry: capturing real-world outcomes of paired vagus nerve stimulation in the chronic stroke population

**DOI:** 10.3389/fstro.2026.1751659

**Published:** 2026-03-31

**Authors:** Shannon J. Doherty, Cecília N. Prudente, Reema Adham Hinds, David Pierce, Navzer D. Engineer, John Carrithers, Diana Hansen, W. Brent Tarver

**Affiliations:** MicroTransponder, Inc., Austin, TX, United States

**Keywords:** clinical trials as topic, neuromodulation, rehabilitation, stroke, upper extremity, vagus nerve stimulation

## Abstract

**Objective:**

To capture real-world use and outcomes from chronic stroke survivors with arm and hand impairment implanted with a paired vagus nerve stimulation (Paired VNS) device. The FDA-approved Vivistim Paired VNS System combines VNS with upper limb rehabilitation to reduce upper-extremity motor deficits and improve function in chronic stroke survivors.

**Design:**

Observational, postmarket patient registry collecting outcomes data from individuals who receive Vivistim for stroke recovery. Assessments will be conducted at baseline and at scheduled assessment timepoints over a period of up to 3 years.

**Participants:**

Adults aged 18 years or older with a history of stroke with moderate-to-severe upper limb deficits and clinically evaluated as appropriate candidates for Paired VNS Therapy.

**Outcome measures:**

Motor outcome assessments include the Fugl-Meyer Assessment-Upper Extremity, the Nine Hole Peg Test, and the Wolf Motor Function Test (optional). Patient-reported outcome assessments include a global quality of life questionnaire, Stroke Impact Scale, Motor Activity Log, and Beck Depression Inventory.

**Conclusions:**

The registry is designed to provide real-world evidence on the use and outcomes of Paired VNS. These data will characterize functional changes, patient experiences, and therapy utilization in a heterogeneous chronic stroke population, supporting optimization and broader integration of Paired VNS into stroke recovery pathways.

**Clinical trial registration:**

https://clinicaltrials.gov/study/NCT05301140, identifier NCT05301140.

## 1 Background

Implantable vagus nerve stimulation (VNS) is a well-established form of neuromodulation used to treat a range of neurological conditions, including intractable epilepsy and depression (Aaronson et al., 2017; Englot et al., 2016). In Paired VNS, stimulation is delivered in conjunction with specific, repeated motor tasks. Repeated pairing of brief bursts of VNS with active, task-specific practice strengthens neural connections that are active during the task, thereby facilitating long-lasting, targeted plasticity.

The mechanism of action of Paired VNS has been previously described but, briefly, stimulation of the vagus nerve activates deep brain nuclei that release neuromodulators, including norepinephrine, acetylcholine, and serotonin (Engineer et al., 2019; Khodaparast et al., 2014). When paired with meaningful tasks, VNS leverages the brain's ability to strengthen neural pathways and relearn impaired motor functions, making it a valuable therapeutic intervention for stroke recovery. In preclinical stroke models, VNS paired with motor training consistently improved strength and motor control compared with task-practice alone and enhanced stable neural plastic changes in the motor cortex (Khodaparast et al., 2014, 2013; Meyers et al., 2018). In people with stroke-related motor impairment, VNS paired with upper limb rehabilitation is safe and effective in improving motor function (Dawson et al., 2016; Kimberley et al., 2018). In a randomized trial of 20 adults with chronic stroke assigned to VNS with rehabilitation or rehabilitation alone, the VNS group showed a clinical trend toward improved impairment and function. In a subsequent double-blind, randomized, sham-controlled trial of 17 participants with chronic stroke, the Paired VNS group had significantly greater improvements in impairment and function at both 30- and 90-days post-therapy compared with those who received task-practice alone.

There were no serious adverse events in either group (Kimberley et al., 2018). Notably, participants from this trial continued Paired VNS Therapy beyond the in-clinic period and maintained their improvements or continued to improve over a subsequent 3 year follow up period. Following the pilot studies, a randomized, triple-blinded, sham-controlled pivotal trial (VNS-REHAB) was conducted with 108 participants with moderate to severe arm impairment after chronic ischemic stroke (Dawson et al., 2021). All participants were implanted with the Vivistim Paired VNS System and randomized to active (Paired VNS) or control (sham VNS) during 18 sessions of upper limb rehabilitation over 6 weeks followed by 3 months of self-activated Paired VNS at home. At the end of the blinded phase, the active VNS group had greater improvement in upper limb impairment, function, and quality of life measures compared with the control group (Dawson et al., 2021; Kimberley et al., 2025). One control subject experienced a temporary vocal cord palsy related to the implantation procedure, which subsequently resolved. Notably, long-term data from the VNS-REHAB trial showed that participants maintained their improvements for at least 1 year, with significant, clinically meaningful gains across multiple domains, including upper limb impairment, daily activity, participation, and quality of life (Kimberley et al., 2025).

Following the successful pivotal trial, the US Food and Drug Administration (FDA) approved the Vivistim Paired VNS System in 2021, with commercial distribution beginning in May 2022. The Vivistim Paired VNS System is intended for use as part of a rehabilitation program in which VNS is paired with high-repetition, task-specific upper limb therapy (Vivistim Paired VNS system; P210007; MicroTransponder Inc., Austin, TX, United States). It is indicated to reduce upper extremity motor deficits and improve motor function in chronic ischemic stroke patients with moderate to severe arm impairment (MRI Safety Information, 2021).

### 1.1 Objective

This article outlines the protocol for the Vivistim Registry for Paired VNS Therapy (GRASP), detailing participant recruitment, data collection, outcome assessments, and planned data analysis. The purpose of the GRASP registry is to gather real-world data on individuals receiving or considering Paired VNS Therapy with Vivistim. Aggregated data will be used to better understand the real-world use and outcomes of Paired VNS Therapy. This protocol was approved by the WCG institutional review board and establishes a framework for future outcomes reporting of the GRASP Registry.

### 1.2 Commercial context of paired VNS therapy

All steps involved in medically evaluating a patient for device eligibility, qualifying for the Vivistim Paired VNS System, obtaining insurance approval, undergoing device implantation, and receiving in-clinic Paired VNS Therapy are conducted as part of the commercial experience and are outside the scope of this registry. The GRASP registry does not alter or dictate any aspect of the treatment pathway. Instead, the registry collects observational, real-world data from patients who are pursuing Paired VNS Therapy as part of their standard care and who voluntarily consent to have their outcomes recorded as they progress through the process. Although the study design is observational, a description of the device and recommended therapy is warranted to provide context.

### 1.3 Device description

The system includes an implantable pulse generator (IPG), lead, and electrode (MRI Safety Information, 2021). Procedural details and safety have been previously described (Liu et al., 2022). The non-implanted system components include a wireless push-button controller, which the therapist uses to trigger VNS during rehabilitation, and the Stroke Application Programming Software (SAPS), which allows the clinician to manage device settings.

Standard commercial device settings are based on the parameters established during clinical trials and include an output current of 0.8 mA, a pulse width of 100 ms, and a stimulation frequency of 30 Hz delivered over 0.5 s (Dawson et al., 2021, 2016; Engineer et al., 2019; Kimberley et al., 2018). Device parameters may be adjusted by a clinician based on individual participant tolerance. Variation in stimulation amplitude was also permitted in previous studies and did not affect efficacy (Dawson et al., 2021, 2016; Engineer et al., 2019; Kimberley et al., 2018).

### 1.4 Paired VNS therapy overview

Paired VNS Therapy comprises both in-clinic (therapist-activated) and out-of-clinic (self-activated) components, wherein VNS is delivered within a therapeutically effective time proximate to functional, goal-directed upper extremity tasks that are meaningful to the individual.

#### 1.4.1 In-clinic therapy

The therapist delivers VNS timed with the patient's active movement attempts during goal-directed tasks. Sessions are typically 90 min and are conducted three times per week for at least 18 sessions. Therapy should be high-intensity and high-repetition (300–500) with tasks that are appropriately challenging and salient to maintain engagement and achieve evidence-based rehabilitation dose and intensity. Tasks might include practicing activities of daily living (self-feeding, dressing) or work and leisure activities (e.g., gardening, wiping surface, playing games) and are assigned based on individual goals and level of impairment. Paired VNS data are recorded in the IPG.

#### 1.4.2 Out-of-clinic therapy

Outside of clinic sessions, individuals can self-activate the device by swiping a magnet over the IPG before performing prescribed functional exercises, activities of daily living, or engaging in hobbies using the affected limb. The magnet swipe activates a 30-min cyclical VNS session with a 0.5-s pulse delivered every 10 s. Magnet settings can be adjusted to allow for 15 min sessions per participant preference, though the system limits self-activation to a maximum of 4 h per 24-h period. Magnet use data are recorded in the IPG.

## 2 Methods

### 2.1 Design

The GRASP registry is an observational postmarket registry designed to collect real-world data on the FDA-approved Vivistim Paired VNS System in individuals with chronic stroke-related upper limb deficits. Most enrolled participants will ultimately receive the device and Paired VNS Therapy. Those who choose not to proceed with implantation may remain in the registry for data collection as a non-implant group, if they choose.

### 2.2 Participants

#### 2.2.1 Inclusion and exclusion criteria

To be enrolled in the registry, participants must meet the following inclusion criteria:

- Have a history of stroke with moderate-to-severe upper limb deficits and be clinically judged to be a good candidate for Paired VNS Therapy.
- Intend to be implanted with the Vivistim System (whether or not they actually do get implanted).
- Be aged 18 years or older.

Individuals will be excluded if they:

- Have not had a stroke.
- Do not have upper limb deficits.
- Are currently enrolled in an investigational study.

### 2.3 Recruitment, consent, withdrawal, and ethical considerations

#### 2.3.1 Recruitment

Individuals who are eligible for the Vivistim System and are interested in undergoing the procedure and Paired VNS Therapy will be invited to participate in the registry. Individuals will typically be approached by the study site coordinator or site principal investigator during the initial medical evaluation visit or in the rehabilitation clinic. Participants will be enrolled at sites across the United States on a rolling basis, with no predefined cap on enrollment. Recruitment will continue unless the sponsor chooses to discontinue data collection for the national registry.

#### 2.3.2 Consent

All participants will provide written informed consent. Potential participants will have ample time to review the consent form and have their questions answered. If the participant needs additional time to consider consenting, the study staff will follow up at a mutually agreed upon time to assess the decision regarding participation. All authorized study personnel, including the principal investigator, study coordinator, and study therapists receive sponsor-provided training on the informed consent process and are qualified to assist potential participants throughout the consent process. Before a participant provides consent, a study staff member will review the registry's purpose and details of registry-related visits. If the individual remains willing to participate, they will sign the consent form, and authorized study personnel will schedule their next visit.

Participants will be apprised of all information collected, and trained study personnel will be available to answer questions or concerns at any time.

#### 2.3.3 Participant retention and reimbursement

Participant retention is supported through alignment of registry assessments with routine clinical visits to minimize additional participant burden. Flexible data collection approaches are permitted, including in-clinic or virtual completion of patient-reported outcome measures and a remote assessment option for FMA-UE. Subject to approval by each site's institutional review board, the registry will compensate participants for in-clinic visits to help defray costs associated with their participation (i.e., transportation and parking). These measures are intended to facilitate continued participation and reduce attrition during the follow-up period. Payments will not be provided for in-clinic therapy sessions as these are part of usual care.

#### 2.3.4 Study withdrawal

Participants can choose to withdraw from the registry at any time. Any registry data collected up to that point will be retained for use in analysis.

#### 2.3.5 Good clinical practices and role of the sponsor

The WCG Institutional Review Board approved the study protocol on February 21, 2022, under tracking identifier 20220693, and the study will be conducted in accordance with the principles outlined in the Declaration of Helsinki. All participants will provide written informed consent prior to participating in the study.

The study sponsor developed this protocol and is responsible for overseeing data collection, management, and analysis. The sponsor has no role in clinical decision-making, outcome assessment, or delivery of rehabilitation therapy. Study investigators include independent clinicians who are directly responsible for patient enrollment and data collection. Publications reporting study outcomes will be led by principal investigators from participating sites. Lead authors will have full access to the study data and will assume primary responsibility for data interpretation, manuscript preparation, and submission.

### 2.4 Registry data

#### 2.4.1 Data collection timeline

Registry data will be collected for up to 3 years, as outlined in Table 1 (additional study forms are included in the Supplementary Data Sheet 1). All participants will contribute baseline information including demographics, medical history, motor assessments, patient-reported outcome measures, and healthcare utilization. For participants who receive the device, baseline data will be collected before the initiation of therapy, which could be prior to implantation and up to and including the day stimulation is activated after implantation. Follow-up data will be collected at 3 and 6 months after Paired VNS is first initiated, and annually for 3 years thereafter. Surgical details such as device serial number and procedure date will be collected for participants who undergo implantation of the Paired VNS System. Persons who enroll but are not implanted may continue to be followed up at the same time points based on their date of registry entry.

**Table 1:** Data collection timeline^*^.

Assessments	Enroll	Baseline	Post implant	End of therapy	3 month	6 month	1 year	2 year	3 year
General documentation									
Consent	X								
Demographics		X							
Baseline rehabilitation assessment		X							
Surgery details			X						
Motor outcome assessments									
FMA-UE		X			X	X	X	X	X
WMFT (optional)		X			X	X	X	X	X
9 HP		X			X	X	X	X	X
Patient-reported outcomes									
Quality of life		X			X	X	X	X	X
SIS		X			X	X	X	X	X
MAL		X			X	X	X	X	X
BDI		X			X	X	X	X	X
Global impression of change					X	X	X	X	X
Other forms									
Healthcare utilization		X					X	X	X
Rehabilitation assessment					X	X	X	X	X
Therapy information				X	X	X	X	X	X
Participant satisfaction survey					X	X	X	X	X
Therapist satisfaction survey					X	X	X	X	X
Satisfaction survey					X	X	X	X	X

9 HP, Nine Hole Peg Test; BDI, Beck Depression Inventory; FMA-UE, Fugl-Meyer Assessment–Upper Extremity; MAL, Motor Activity Log; SIS, Stroke Impact Scale; WMFT, Wolf Motor Function Test.

^*^Demographic information and baseline measures are performed within 30 days of surgery. Three- and 6-month assessments and questionnaires are collected ±15 days of the target date. 1-, 2-, and 3-year assessments and questionnaires are collected ±60 days of the target date.

A physical or occupational therapist will perform baseline rehabilitation assessments and subsequent follow-up therapy evaluations. The assessments will document hand dominance, side of paresis, range of motion, strength, gross motor function, fine motor control, spasticity, sensory function, pain, and activity limitations. Appropriate rehabilitation goals and individual patient goals will also be included.

After completing all in-clinic therapy sessions, additional therapy-related information will be collected, including the total number of in-clinic sessions, the frequency of self-activated sessions, and downloaded IPG data.

Because this registry is observational and does not mandate therapy delivery or assessment adjudication at different sites, no formal primary or secondary outcome hierarchy was prespecified.

#### 2.4.2 Motor outcome assessments

##### 2.4.2.1 Fugl-Meyer assessment–upper extremity (FMA-UE)

The FMA-UE is a measure of upper limb motor impairment that quantifies voluntary motor control in relation to the typical evolving synergy patterns experienced by people with stroke as described by Twitchell (1951). In particular, the FMA-UE assesses components of movement in the shoulder, elbow, forearm, and hand, including active range of motion, strength, coordination, and hypertonia. Each of the 33 tests is scored on a 3-point scale as 0, the task cannot be performed; 1, the task is performed partially; or 2, the task is performed fully. The only exception to this scoring is reflex activity, which is scored 0 (absent) or 2 (present). The maximum score on the FMA-UE is 66 points. The FMA-UE is considered a good measure of motor impairment in stroke research (Sanford et al., 1993) with demonstrated validity, reliability, excellent intra- and interrater consistency, and responsiveness to change. The FMA-UE was the primary outcome measure used in the VNS-REHAB pivotal trial (Dawson et al., 2021).

##### 2.4.2.2 Nine Hole Peg Test

The Nine Hole Peg Test is a standardized, quantitative assessment used to measure finger dexterity. The test is performed by taking pegs from a container, one by one, and placing them into holes on a board as quickly as possible and then removing them again, one by one, and placing them back in the container. The test is scored as the total time in seconds to perform the entire activity (Chen et al., 2009).

##### 2.4.2.3 Wolf Motor Function Test (WMFT)

The WMFT is a quantitative measure of upper limb motor ability. The assessment includes timed movements, strength measures (items 7 and 14), and evaluations of movement quality and functional performance. The quality of performance is scored up to 75 points for 15 tasks, and the timed performance permits a maximum of 120 s per task. The WMFT has excellent test-retest reliability, inter- and intra-rater reliability, and internal consistency (Morris et al., 2001; Whitall et al., 2006; Wolf et al., 2001). It was the secondary outcome measure in the VNS-REHAB pivotal trial and is an optional assessment in the registry as not every clinic site has the ability to perform it (Dawson et al., 2021).

#### 2.4.3 Patient-reported outcome measures

##### 2.4.3.1 Quality of life questionnaire

A standardized questionnaire will be used to assess global quality of life. The questionnaire has five dimensions including mobility, self-care, usual activities, pain/discomfort, and anxiety/depression (Brazier et al., 1993).

##### 2.4.3.2 Stroke Impact Scale

The Stroke Impact Scale is a self-report questionnaire that evaluates disability and health-related quality of life after stroke (Mulder and Nijland, 2016).

##### 2.4.3.3 Motor Activity Log-14

The Motor Activity Log-14 is a semi-structured interview that assesses arm function in real-life situations (Uswatte et al., 2006, 2005).

##### 2.4.3.4 Beck Depression Inventory

The Beck Depression Inventory is a self-report inventory that identifies the overt behavioral characteristics of depression (Aben et al., 2002).

##### 2.4.3.5 Global Impression of Change

The Global Impression of Change [Improvement only, Patient (GIC-P, 7 levels)/Clinician (GIC-C, 5 levels)] is an observer-rated scale that measures symptom change or global improvement (Hurst and Bolton, 2004).

##### 2.4.3.6 Other assessments

The registry also includes custom surveys developed by the sponsor to assess healthcare utilization, work status, and satisfaction.

### 2.5 Data capture and management

All data will be initially recorded on paper forms. Trained study site staff will then enter data into the electronic data capture (EDC) system (EDC system; Simplified Clinical Data Systems, Portsmouth, NH, United States) provided by the sponsor. Participants' names, addresses, phone numbers, and email addresses will not be entered into the EDC system. Instead, each participant will be assigned a unique study ID number. A file linking participant ID numbers with names and contact information will be maintained by the site registry coordinator in a password-protected file, which will not be shared with the study sponsor.

Patient-reported outcome forms will either be emailed to participants or completed on paper during study site visits. These hard copies will then be entered into the EDC by study staff. Original paper forms will be securely stored at the clinical site in a locked environment. Adverse event information will not be collected as part of the GRASP registry but will be documented in the standard clinical records within the electronic healthcare record.

The EDC system uses cloud-based Azure data software (Microsoft Inc., Redmond, WA, United States). All data entered into the EDC (a customer data platform, or CDP) are encrypted and stored in data centers (Microsoft Inc.) that are monitored continuously and are fully compliant with applicable regulations. The system is password protected and study data are accessible only by authorized users who have been trained on the CDP. The EDC system is centrally managed with role-specific data protection and row-level security. All personnel are given their own passwords and do not share passwords with anyone else. The EDC system meets the compliance standards and certifications for handling protected health information.

#### 2.5.1 Data availability

Contributing sites and principal investigators retain access to their own site-level data and can receive access to anonymized summary analyses on all aggregated data upon request. Sites will not have access to other sites' individual-level data. Publication of registry data, other than individual site data, is not permitted without the advance written approval of the study sponsor and the other clinicians or principal investigators contributing to the dataset.

#### 2.5.2 Data monitoring

Data collected in this registry will be monitored as outlined in the study monitoring plan. Adverse events and safety information will be captured through standard postmarket surveillance and regulatory medical device reporting processes but will not be collected or reported via the registry.

#### 2.5.3 Provisions to protect the privacy interests of participants

On-site data collection for the registry should occur in a private clinical space without interference from other staff or patients. Participants can choose whether to transmit survey questionnaires via email or whether to complete them in person during in-clinic visits. Participants will be made aware that some information for the registry will be collected from their therapist and surgeon or surgeon's staff and recorded under a unique study identifier to protect their privacy. As necessary for collecting such information, the participant will be asked to sign a standard release of information form. Some health-related data or measures collected as part of usual care may be obtained directly from the participant's electronic medical record. Participants may ask questions at any time throughout their registry participation, including information about scores on outcome measures collected.

#### 2.5.4 Protected health information

This study will include access to participants' medical records to obtain medical background information, therapy information, and registry-related outcome measures collected as part of usual care. Therefore, HIPAA (Health Insurance Portability and Accountability Act) language will be included in the consent form.

### 2.6 Data analysis

All registry data will be summarized with descriptive statistics (mean, median, range, and SD) for continuous data and counts and percentages for categorical data at each scheduled timepoint assessment. No minimum number of therapy sessions or device usage is required for inclusion in descriptive analyses. Established minimal clinically important difference (MCID) thresholds for motor and patient-reported outcomes will be used to aid interpretation, where applicable. The approach to handling missing data will be determined based on the type and extent of the missing data, with specific models to be defined prior to formal data analysis.

IPG usage data are downloaded at the completion of in-clinic therapy and at each subsequent in-clinic follow-up visit through 3 years. Study forms instruct sites to download IPG data at each applicable post-therapy visit. If a scheduled download is missed, data remain stored on the device and may be retrieved at a subsequent visit. Missing or unavailable device data will be documented and summarized descriptively.

Additional exploratory analyses to supplement the results or for research may be performed, as appropriate.

### 2.7 Changes to methods

The exclusion criteria were modified from a previous protocol version to allow retrospective enrollment for patients who consent after implantation. There are no timeline restrictions. This change was made to maximize data capture in the registry, as some sites were performing commercial implants prior to formally participating as study sites.

## 3 Results

### 3.1 Current status

The first GRASP registry site was activated in December 2022, and the first participant enrolled in April 2023. Active registry sites encompass a broad range of care settings, including academic medical centers, community hospitals, outpatient rehabilitation clinics, private practices, and mobile therapy providers. As of January 2026, there are over 60 active sites across 39 states with over 200 participants enrolled and 150 implanted with the Vivistim Paired VNS System. A non-implant group is not currently available, as all enrolled participants have either received the device or are awaiting implantation. Enrollment remains ongoing.

Future publications reporting on registry outcomes will be led by principal investigators or participating site clinicians comprising the GRASP Registry authorship group to minimize potential bias. Lead authors will have access to registry data and will assume primary responsibility for data interpretation, manuscript preparation, and submission.

## 4 Discussion

Upper limb paresis is one of the most common and persistent consequences of stroke, with up to 65% of people unable to fully incorporate their affected limb into daily activities by 6 months post-stroke (Dobkin, 2005). These enduring upper limb deficits are a leading contributor to long-term disability, diminishing a person's quality of life and limiting participation in meaningful activities (Lieshout et al., 2020). The Vivistim Paired VNS System is an approved treatment for chronic stroke upper limb recovery that has been shown to reduce motor deficits, improve functional outcomes, and enhance engagement and quality of life (Dawson et al., 2021; Kimberley et al., 2025). The GRASP registry extends this evidence by systematically collecting real-world outcomes from individuals receiving VNS paired with rehabilitation therapy. These data will enable a deeper understanding of patient characteristics and experiences, therapy delivery, and clinical practice.

### 4.1 Limitations

The observational design of this registry precludes causal inference, and the open-label delivery of commercially available Paired VNS Therapy may introduce selection or expectation biases. To address these limitations, analyses will primarily be descriptive, emphasizing characterization of real-world outcomes, practice patterns, and variability in therapy utilization. Where feasible, outcomes may be explored and stratified by baseline characteristics, therapy exposure, or site-level factors to better contextualize findings.

Variability in clinical practice across sites may make direct comparisons and interpretation of outcomes more challenging. However, this heterogeneity is an inherent and valuable feature of real-world evidence. By capturing data across diverse clinical environments, the registry enables evaluation of differences in care delivery, resources, and outcomes in routine practice. This approach complements rigorously controlled clinical trial data by providing insight into real-world implementation and utilization.

The extended duration of follow-up may increase the likelihood of participant attrition and missing data. While participant retention strategies are employed, missing data will be evaluated based on the extent and pattern of missingness and addressed using appropriate analytic approaches, with results interpreted accordingly by principal investigators.

The Vivistim System is currently approved and commercially available in the U.S. only. As such, the GRASP registry is designed to collect real-world evidence relevant to U.S. clinical practice, and findings are therefore most directly applicable to healthcare systems with similar access to surgical implantation and rehabilitation infrastructure.

## 5 Conclusion

The primary purpose of the GRASP registry is to provide real-world use and outcomes data on VNS paired with upper-limb rehabilitation. Analysis of registry data will provide valuable insights into how Paired VNS Therapy affects arm and hand function in a heterogeneous real-world population living with chronic post-stroke motor impairments. These findings will expand the real-world evidence base for Paired VNS and help bridge the gap between research and practice by supporting generalizability and integration into standard-of-care stroke recovery pathways.
