# Supplementary material for: Study protocol for the Vivistim GRASP registry: capturing real-world outcomes of paired vagus nerve stimulation in the chronic stroke population
Source: Front Stroke. 2026 Mar 31;5:1751659. doi: 10.3389/fstro.2026.1751659 (PMC13076104; doi:10.3389/fstro.2026.1751659)
Supplement: Supplementary file 1 [file Data_Sheet_1.pdf]

Demographics and Medical History

1. **Gender**: (Choose one.) ☐ Male ☐ Female ☐ Other (non-binary)
2. **Date of birth:** ________________ **(MM/DD/YYYY)**
3. **Ethnicity** (Choose one with which you MOST CLOSELY identify):
   ☐ Hispanic or Latino ☐ Not Hispanic or Latino ☐ Unknown ☐ Not reported
4. **Race** (Choose all those with which you identify):
   ☐ American Indian or Alaska Native ☐ Asian
   ☐ Black or African-American ☐ Native Hawaiian or Other Pacific Islander
   ☐ White ☐ Unknown ☐ Not reported
5. **Dominant Hand:** ☐ Right hand ☐ Left hand ☐ Both hands ☐ Unknown
6. **Current smoker?** (Smoked within the past year) ☐ Yes ☐ No ☐ Unknown

Has a doctor or other medical professional ever told an immediate family member (i.e., parent, sibling, child) that he/she has or has had the following?

7. **Stroke:** ☐ Yes ☐ No ☐ Unknown
   a. **Youngest age immediate family member suffered a stroke:** _____ years ☐ Unknown
   b. **Ischemic stroke:** ☐ Yes ☐ No ☐ Unknown
   c. **Hemorrhagic stroke:** ☐ Yes ☐ No ☐ Unknown
8. **Brain aneurysm:** ☐ Yes ☐ No ☐ Unknown
9. **Migraines:** ☐ Yes ☐ No ☐ Unknown
10. **Coronary artery disease:** ☐ Yes ☐ No ☐ Unknown
    (e.g., heart attack)
    If **YES**, youngest age immediate family member was diagnosed with condition / suffered a heart attack: _____ years
11. **Epilepsy:** ☐ Yes ☐ No ☐ Unknown

Has a doctor or other medical professional ever told you that you have or have had the following?

12. **Any stroke:** ☐ Yes ☐ No ☐ Unknown
    (If No or Unknown, no enrollment)
    a. **Ischemic stroke:** ☐ Yes ☐ No ☐ Unknown
       i. Number of ischemic strokes: ☐ None ☐ 1 ☐ ≥ 2 ☐ Unknown
       ii. Recency of ischemic strokes: ____________ Date of most recent stroke ☐ Unknown

Demographics and Medical History

b. **Hemorrhagic stroke :** ☐ Yes ☐ No ☐ Unknown

If **YES**, indicate type(s): ☐ Intracerebral hemorrhage (ICH) ☐ Subarachnoid hemorrhage (SAH) ☐ Hemorrhage unspecified ☐ Unknown

13. **Transient ischemic attack (TIA):** ☐ Yes ☐ No ☐ Unknown

14. **Arteriovenous malformation (AVM):** ☐ Yes ☐ No ☐ Unknown

15. **Migraine(s):** ☐ Yes ☐ No ☐ Unknown

If **YES**, migraine(s) with aura: ☐ Yes ☐ No ☐ Unknown

16. **Carotid endarterectomy:** ☐ Yes ☐ No ☐ Unknown

If **YES**, indicate location: ☐ Left side ☐ Right side ☐ Both ☐ Unknown

17. **Carotid artery stenting**: ☐ Yes ☐ No ☐ Unknown

If **YES**, indicate location: ☐ Left side ☐ Right side ☐ Both ☐ Unknown

Has a doctor or other medical professional ever told you that you have or have had the following?

18. **Seizure episode:** ☐ Yes ☐ No ☐ Unknown

19. **Epilepsy/ Seizure disorder:** ☐ Yes ☐ No ☐ Unknown

20. **Current clinical depression:** ☐ Yes ☐ No ☐ Unknown

21. **Head trauma:** ☐ Yes ☐ No ☐ Unknown

If **YES**, indicate if head trauma resulted in any of the following: (Choose all that apply.)

☐ Loss of consciousness > 30 minutes ☐ Post traumatic amnesia > 24 hours

☐ Abnormal brain imaging findings ☐ None of the above

22. **Atrial fibrillation (AF) / flutter:** ☐ Yes ☐ No ☐ Unknown

a. **Rheumatic heart disease:** ☐ Yes ☐ No ☐ Unknown

b. **Other cause of AF:** ☐ Yes ☐ No ☐ Unknown

If **YES**, specify other cause: ______________________________

23. **Coronary artery disease:** ☐ Yes ☐ No ☐ Unknown

Demographics and Medical History

Has a doctor or other medical professional ever told you that you have or have had the following?

24. Myocardial infarction: ☐ Yes ☐ No ☐ Unknown

25. Angina: ☐ Yes ☐ No ☐ Unknown

26. Cardiac surgery: ☐ Yes ☐ No ☐ Unknown

a. Indicate type(s): ☐ Coronary artery bypass graft (CABG)

☐ Cardiac valve surgery, including non-open surgery (i.e., percutaneous valvuloplasty)

☐ Other, specify: ____________________

b. Date of most recent cardiac surgery: ____________________

27. Coronary stent or PTCA: ☐ Yes ☐ No ☐ Unknown

28. Congestive heart failure: ☐ Yes ☐ No ☐ Unknown

29. Diabetes mellitus: ☐ Yes ☐ No ☐ Unknown

a. Age diabetes first diagnosed: ______ **years**

b. Complications of diabetes (Choose all that apply.): ☐ Nephropathy ☐ Neuropathy ☐ Retinopathy ☐ Other, specify: ____________________ ☐ None of the above

c. Treatment for diabetes (Choose all that apply.)**:** ☐ Diet ☐ Oral medication ☐ Insulin ☐ None of the above

Has a doctor or other medical professional ever told you that you have or have had the following?

30. High blood cholesterol / Hypercholesterolemia: ☐ Yes ☐ No ☐ Unknown

If **YES**, indicate treatment(s) (Choose all that apply.): ☐ Diet ☐ Statins ☐ Other medicines ☐ None of the above

31. Cancer: ☐ Yes ☐ No ☐ Unknown

a. Type(s) of cancer: ☐ Brain ☐ Breast ☐ Colorectal ☐ Endometrial ☐ Esophagus ☐ Lung ☐ Prostate ☐ Renal (kidney) ☐ Skin ☐ Other, specify: ____________________

b. Did you receive head or neck radiation to treat the cancer?
☐ Yes ☐ No ☐ Unknown

Demographics and Medical History

Answer the following items on this form about the participant's/subject's functional status *after the stroke event.*

32. Ambulatory status:
- ☐ Able to ambulate independently (no help from another person) w/ or w/o device
- ☐ Able to ambulate with assistance (from person)
- ☐ Unable to ambulate
- ☐ Unknown

Previous Rehabilitation

33. Type(s) of rehabilitation (rehab) services received (Choose all that apply):
- ☐ Received rehab services during hospitalization
- ☐ Referred to rehab services following discharge
- ☐ Ineligible to receive rehab services due to impairment
- ☐ Ineligible to receive rehab services /insurance issues
- ☐ Home health with PT/OT/etc.
- ☐ Other, specify ________________
- ☐ Transferred to rehab facility
- ☐ Ineligible to receive rehab because symptoms resolved
- ☐ Received rehab in skilled nursing facility
- ☐ Outpatient rehab only

34. Type(s) of rehabilitation therapy disciplines received and month/year (Choose all that apply):
- ☐ Speech/ Language __/__
- ☐ Occupational __/__
- ☐ Vocational __/__
- ☐ Physical __/__
- ☐ Psychological __/__
- ☐ Dietary __/__
- ☐ Recreational __/__
- ☐ Other, specify ______________________________ __/__

Answer the following items on the subject's prior upper limb rehabilitation

35. Approximate Frequency of rehabilitation therapy: ______ visits in ______weeks

36. Average duration of rehabilitation therapy visit: ______ minutes (within ~15-minute approximation)

37. Setting for rehabilitation therapy: ☐ Inpatient ☐ Outpatient ☐ Home health

38. Do you still receive rehabilitation? ☐ Yes ☐ No

Demographics and Medical History

Follow-up Care

1. Date of original stroke: _____________ Date of discharge from original stroke: _________________
2. Which side of the brain is the stroke lesion located? ☐ Left ☐ Right ☐ Both
3. What is the most paretic hand? ☐ Left ☐ Right
4. Follow-up care from stroke specialist? ☐ Yes ☐ No
5. Follow-up care from primary care physician? ☐ Yes ☐ No
6. Follow-up care from rehabilitation doctor? ☐ Yes ☐ No ☐ Not applicable

Completed by: ____

Name Signature Date

Investigator: ____

Name Signature Date

SUBJECT ID ____________________

DATE ________________________

Baseline Rehabilitation Assessment

Paretic Arm (circle one): R or L **Dominant Arm (circle one):** R or L

Significant Medical Issues:

____

____

____

____

Pain

Shoulder: ☐ Absent ☐ Present Level (0-10): ____________

Hand/Wrist: ☐ Absent ☐ Present Level (0-10): ____________

Arm (other): ☐ Absent ☐ Present Level (0-10): ____________

Other (not arm): ☐ Absent ☐ Present (Describe location and level (0-10)): ____________________

____

____

UE Impairment Summary (Notes on ROM, Strength, Gross motor function, Fine motor control, Spasticity, Sensory function, and Limitations in Activity/Participation)

____

____

____

____

____

____

____

____

____

____

Baseline Rehabilitation Assessment

Subject Stated Goal(s):

#1: ____

#2: ____

#3: ____

Rehabilitation Goals:

#1: ____

#2: ____

#3: ____

Final Impressions and Notes:

____

____

____

____

____

____

____

Completed by: ____
Name Signature Date

Investigator: ____
Name Signature Date

Surgery Details Form

Surgery Date: ____________________ (MM/DD/YYYY)

MTI Lead Model number_______ Serial Number __________ Size ____________

MTI IPG Model number _______ Serial number __________

Lead Test

i) **Was lead test conducted:** ☐ Yes ☐ No

ii) **Lead impedance:** ☐ OK, Value ________ ☐ >10k (high)

iii) **Implant battery:** ☐Green ☐Other

iv) **Notes:**____

____

____

Surgeon (Print Name): _________________________

Were there any adverse events due to the surgery? ☐ No ☐ Yes (explain below)

Were there any serious adverse events due to the surgery? ☐ No ☐ Yes (explain below)

Please note any other relevant information about the surgery (different anatomy, any difficulties or anomalies, local/regional anesthesia, etc.):

____

____

____

Completed by: ____
Name Signature Date

Investigator: ____
Name Signature Date

Therapy Details – End of Therapy

1. **Information about in-clinic rehabilitation sessions:**
   a. Start Date: ____/____/______ (MM/DD/YYYY)
   b. End Date: ____/____/______ (MM/DD/YYYY)
   c. Number of in-clinic Therapy Sessions ________
   d. Typical length of in-clinic Therapy Sessions ________ minutes
   e. Device settings ☐**Standard (1/2 Sec, 0.8 mA, 100 uS)** ☐**Other __ Sec, __ mA, __ uS**

2. **Information about at-home magnet use:**
   a. Does the patient do exercises at home during stimulation? ☐ **Yes** ☐ **No**
   b. Have you verified magnet use using the Vivistim SAPS software? ☐ **Yes** ☐ **No**
   c. How long is each at-home session and how many times a day (on average) does the patient do at-home exercises and VNS?

____

____

   d. What types of exercises does the patient do at home?

____

____

____

3. **Please download the statistics from the IPG.** ☐ **Completed** ☐ **Not Done (explain below)**

____

____

Completed by: ____
Name Signature Date

Investigator: ____
Name Signature Date

Therapy Details – Follow-Up Visits

Visit (Check One): ☐ 3mo ☐ 6mo ☐ 12mo ☐ 2y ☐ 3y ☐ Not Done

1. Information about in-clinic rehabilitation sessions:

a. Start Date: ____/____/______ (MM/DD/YYYY)

b. End Date: ____/____/______ (MM/DD/YYYY)

c. Number of in-clinic Therapy Sessions ________

d. Typical length of in-clinic Therapy Sessions ________ minutes

e. Device settings ☐**Standard (1/2 Sec, 0.8 mA, 100 uS)** ☐**Other __ Sec, __ mA, __ uS**

2. Information about at-home magnet use:

a. Does the patient do exercises at home during stimulation? ☐ **Yes** ☐ **No**

b. Have you verified magnet use using the Vivistim SAPS software? ☐ **Yes** ☐ **No**

c. How long is each at-home session and how many times a day (on average) does the patient do at-home exercises and VNS?

____

____

d. What types of exercises does the patient do at home?

____

____

____

3. Please download the statistics from the IPG. ☐ **Completed** ☐ **Not Done (explain below)**

____

____

Completed by: ____

Name Signature Date

Investigator: ____

Name Signature Date

SUBJECT ID ________________

DATE ________________

Satisfaction Survey

Visit (Check One): ☐ 3mo ☐ 6mo ☐ 12mo ☐ 2y ☐ 3y ☐ Not Done

Check one response to each question.

1. **Overall, how satisfied are you with your Vivistim Paired VNS Therapy (the specific stimulation treatment you have received so far from your implant for your movement deficits)?**

   ☐ Very satisfied ☐ Satisfied

   ☐ Neither satisfied nor dissatisfied

   ☐ Dissatisfied ☐ Very dissatisfied

2. **Overall, how satisfied are you with your overall health since you started Vivistim Paired VNS Therapy?**

   ☐ Very satisfied ☐ Satisfied

   ☐ Neither satisfied nor dissatisfied

   ☐ Dissatisfied ☐ Very dissatisfied

3. **How likely are you to recommend Vivistim Paired VNS Therapy to a friend or family member experiencing arm and hand impairment after stroke?**

   ☐ Very Likely ☐ Likely ☐ Somewhat Likely ☐ Not Likely

4. **How satisfied are you with your ability to tolerate any side effects you associate with your device?**

   ☐ Very satisfied ☐ Satisfied

   ☐ Neither satisfied nor dissatisfied

   ☐ Dissatisfied ☐ Very dissatisfied

5. **How satisfied are you with your progress toward the primary goal(s) agreed upon with your Therapist at the beginning of treatment?**

   ☐ Very satisfied ☐ Satisfied

   ☐ Neither satisfied nor dissatisfied

   ☐ Dissatisfied ☐ Very dissatisfied

I've noticed improvements in (check all that apply): ☐ Arm Function ☐ Hand Function ☐ Speech ☐ Memory/Thinking Ability ☐ Walking ☐ Balance ☐ Mood ☐Other __________ (Please Describe)

My main goals for improvement with the Vivistim System are:

1 - ____

2 - ____

3 - ____

Reviewed by: ____

Name Signature Date

Investigator: ____

Name Signature Date

SUBJECT ID ____________________

DATE ________________________

GLOBAL IMPRESSION OF CHANGE

Visit (Check One): ☐ 3mo ☐ 6mo ☐ 12mo ☐ 2y ☐ 3y ☐ Not Done

Participant: Ask the following question to the subject:

Since I enrolled in the study, my overall arm and hand function is:

o Very Much Improved

o Much Improved

o Minimally Improved

o No Change

o Minimally Worse

o Much Worse

o Very Much Worse

Therapist: Answer the question below:

In your opinion as a clinician, compared to the subject's situation at enrollment, would you say his/her arm and hand function is:

o Much Better

o Slightly Better

o About the Same

o Slightly Worse

o Much Worse

Reviewed/Completed by: ____
Name Signature Date

Investigator: ____
Name Signature Date

Healthcare Utilization & Work Status

Visit (Check One): ☐ **Baseline** ☐ **12mo** ☐ **2y** ☐ **3y** ☐ **Not Done**

Healthcare Utilization

1. **How many times and for what reason has the subject visited the following facilities *in the last 3 months* for impairments, injuries and/or complications *related to stroke*?**

   A. Rehabilitation Outpatient: ____________ visits Reason: ____________________
   B. Clinic: ____________ visits Reason: ____________________
   C. Urgent Care: ____________ visits Reason: ____________________
   D. Emergency Room: ____________ visits Reason: ____________________
   E. Hospital Outpatient: ____________ visits Reason: ____________________
   F. Hospital Inpatient (overnight stay): ____________ visits Reason: ____________________
   G. Other (specify _______________): ____________ visits Reason: ____________________
   H. None

Work Status

2. **Is the subject currently working?**

   ☐ No (Skip #3 & #4) ☐ Yes, part time ☐ Yes, full time

3. **Did the subject's productivity improve after Vivistim therapy?**

   ☐ No ☐ Yes ☐ N/A (for baseline)

4. **If the subject is currently working, has he/she missed any work days *in the past month* because of the paretic arm/hand?**

   ☐ No ☐ Yes If yes, how many days did you miss? ____________

Completed by: ____
Name Signature Date

Investigator: ____
Name Signature Date

Rehabilitation Assessment – Follow-up

Visit (Check One): ☐ 3mo ☐ 6mo ☐ 12mo ☐ 2y ☐ 3y ☐ Not Done

Paretic Arm (circle one): R or L **Dominant Arm (circle one):** R or L

Changes in Medical Issues (check one): ☐ No ☐ Yes (Describe below)

____

____

____

____

Pain

Shoulder: ☐ Absent ☐ Present Level (0-10): __________

Hand/Wrist: ☐ Absent ☐ Present Level (0-10): __________

Arm (other): ☐ Absent ☐ Present Level (0-10): __________

Other (not arm): ☐ Absent ☐ Present (Describe location and level (0-10)): ______________

____

____

Updates in UE Impairment (Notes on ROM, Strength, Gross motor function, Fine motor control, Spasticity, Sensory, and Limitations in Activity/Participation)

____

____

____

____

____

____

____

____

____

Rehabilitation Assessment – Follow-up

Refer to baseline goals for this section

Subject Stated Goal(s) Status (check one):

#1: ☐ Met / ☐ Unmet, comment: ____

#2: ☐ Met / ☐ Unmet, comment: ____

#3: ☐ Met / ☐ Unmet, comment: ____

Rehabilitation Goals Status (check one):

#1: ☐ Met / ☐ Unmet, if unmet, reason: ___________________________________

#2: ☐ Met / ☐ Unmet, if unmet, reason: ___________________________________

#3: ☐ Met / ☐ Unmet, if unmet, reason: ___________________________________

Home Exercise Program (HEP) [PLEASE DISREGARD QUESTIONS ABOUT HEP]:

HEP Issued: ☐ No ☐ Yes, no changes ☐ Yes, with updates (Attach copy)

Approximate number of magnet swipes since last visit: ______________

Subject Reported Compliance with HEPs [PLEASE DISREGARD QUESTIONS ABOUT HEP]

☐ As prescribed ☐ More than prescribed ☐ Less than prescribed ☐ Non-compliant

If non-compliant, please explain:____

Final Impressions and Notes:

____

____

____

Log File:

1. Read Statistics: ☐Completed ☐Not completed
2. Export Log: ☐ Completed ☐Not completed

Completed by: ____
Name Signature Date

Investigator: ____
Name Signature Date

Therapist Satisfaction Survey

Visit (Check One): ☐ 3mo ☐ 6mo ☐ 12mo ☐ 2y ☐ 3y ☐ Not Done

1. Overall, how satisfied are you with the Paired VNS therapy sessions?

☐ Very Satisfied ☐ Satisfied ☐ Neither Satisfied nor Dissatisfied

☐ Dissatisfied ☐ Very Dissatisfied

2. How easy was it to guide the therapy session while simultaneously triggering VNS?

☐ Very Difficult ☐ Difficult ☐ Neutral ☐ Easy ☐ Very Easy

3. How satisfied were you with the Paired VNS therapy training (excludes assessment training)?

☐ Very Satisfied ☐ Satisfied ☐ Neither Satisfied nor Dissatisfied

☐ Dissatisfied ☐ Very Dissatisfied

4. How easy was it to learn to use the Vivistim Paired VNS system in a therapy session?

☐ Very Difficult ☐ Difficult ☐ Neutral ☐ Easy ☐ Very Easy

5. How likely are you to recommend Paired VNS therapy as a treatment method to your colleagues?

☐ Very Likely ☐ Likely ☐ Not Very Likely ☐ Not Likely At All

6. How likely are you to recommend the Paired VNS therapy to your clinically appropriate patients?

☐ Very Likely ☐ Likely ☐ Not Very Likely ☐ Not Likely At All

Therapist Satisfaction Survey

7. The average length of the therapy sessions was (circle one):

60 min 90 min 120 min Other: __________

8. The subject's ability to tolerate the sessions was:

☐ Very Difficult ☐ Difficult ☐ Just Right ☐ Easy ☐ Very Easy

9. How difficult was it to plan and implement for therapy sessions longer than 30-45 min?

☐ Very Difficult ☐ Difficult ☐ Neutral ☐ Easy ☐ Very Easy

10. Is there anything else you would like to add about Vivistim and Paired VNS therapy?

____

____

____

____

____

____

____

____

____

Completed by: ____

Name Signature Date

Investigator: ____

Name Signature Date
